# Application of Kano model for optimizing the training system among nursing internship students: a mixed-method Egyptian study

**DOI:** 10.1186/s12912-023-01485-5

**Published:** 2023-09-14

**Authors:** Ahmed Abdelwahab Ibrahim El-Sayed, Sally Mohammed Farghaly Abdelaliem

**Affiliations:** 1 Nursing Administration Department, PhD, MSN in Nursing Administration, BSc in Nursing Sciences, Lecturer of Nursing Administration, Faculty of Nursing, Alexandria University, Alexandria, RN Egypt; 2 Nursing Administration Department, PhD, MSN in Nursing Administration, BSc in Nursing Sciences, Associate Professor of Nursing Administration, Faculty of Nursing, Alexandria University, Alexandria, RN, FEHA Egypt

**Keywords:** Kano model application, Training system, Nursing, Internship students, Evidence-based, Egyptian study

## Abstract

**Background:**

Clinical experience is an important component of nursing education because it translates students' knowledge into practice, which serves as the cornerstone of nursing practice in health care delivery.

**Purpose:**

The study aims to explore the quality attributes required for optimizing the training system of nursing internship students using Kano model.

**Methods:**

A concurrent exploratory sequential triangulation design was used for mixed-methods research. A total of 295 nursing internship students (Target Population) were recruited (whole-population sampling) from the study settings in Egypt. Of them, 280 (97.2%) agreed to participate in the study and completed the interview and the self-administered questionnaire. Data collection was done over 6 months from February to August, 2022. Inferential statistics and thematic data analysis were used to analyze the results.

**Results:**

Findings revealed that there were 35 fundamental attributes required for high-quality nursing students’ internship training. Kano model was used to categorize and prioritize the 35 quality attributes. Kano analysis revealed that 22 attributes were categorized as "attractive" and 11 attributes were as categorized as "must be" and two were indifferent attributes.

**Conclusion:**

Incorporating the voice of nurse interns during their training is the key to providing efficient and high-quality internship training experience. It could give realistic impressions about the drawbacks of training and proposed solutions.

**Implications of the study:**

Nurse managers and educators in clinical settings and educational institutions should put much emphasis on the training attributes and pillars to ensure that nursing internship students are mastering the skills of competent alumni. Provision of conducive training environment that fulfill the basic needs of internship students to maintain passion for learning as well as commitment of internship students to nursing profession will improve the satisfaction level and quality of education, training, and practice. Also, incorporating internship students support system with motivation strategies are helpful tools to maintain exemplary performance of internship students during the training period.

**Supplementary Information:**

The online version contains supplementary material available at 10.1186/s12912-023-01485-5.

## Introduction

Quality has emerged as a hot topic in recent years, not only in the field of manufacturing but also in a variety of service industries. In the field of higher education, quality is also given a lot of consideration, and these days, a wide range of themes can be found that are researched from both a practical and a scientific point of view. The prevailing idea nowadays is that a person who uses higher education institutions' products—knowledge and skills obtained there—is a "customer" of those institutions. Thus, a student is a higher education institution's principal (direct) client, and their employer is its secondary (indirect) client [1]. Investigations into the quality of internship clinical training have frequently focused on nursing internship students, and care has been taken to understand them as well as to identify their needs and expectations.

Globally, there is a continuing scarcity of registered nurses, with a high turnover rate. This scarcity is the result of educational, systemic, societal, and individual factors. Clinical experience, in particular, is an important component of nursing education because it translates students' knowledge into practice, which serves as the cornerstone of nursing practice in health care delivery [2]^.^ However, it is clear from the nursing literature that high turnover rates, particularly among new graduate nurses, are the result of inadequate training, a lack of support structures, and significant stress in the workplace [3].

The most important aspect influencing nursing students' clinical learning experiences is the interaction between staff and nursing internship students, since this promotes the learning process in clinical settings [2]. It is critical that each member of the healthcare team contribute to delivering a pleasant clinical experience that fosters the improvement of nursing students' clinical competence [3]. Nursing schools must be cautious in protecting students' experiences in order to effectively accomplish clinical objectives and bridge the gap between theory and practice [4].

Nursing internship students are recent graduates with little real-world job experience. These newcomers are working to improve their skills in providing great nursing care under the supervision of preceptors. As a result, this study looked into the obstacles and difficulties that nursing students encountered throughout their internship year. Knowledge of the hurdles that may impede their learning in the clinical context is necessary to assist nurse educators and clinical staff in developing suitable measures to enable the progression of nursing internship students' clinical competence [1].

Organizations that offer educational and training services are currently experiencing a significant issue connected to the competitiveness of their offer, which is the driving force behind the selection of this research topic. In a market economy that allows for the extensive development of educational institutions, the competition for each customer is waged on a variety of fronts, including the variety of services provided, the setting and manner in which they act, the caliber of their message, and interpersonal relationships. Additionally, it should be highlighted that due to their specialization, education and clinical training hold a special place in the catalog of service kinds. This is due to the fact that learning and training as a whole take time, yet their efficacy can only be determined afterward [5–7]. Long-term, nearly every area of social life is impacted by the quality of educational services [7]. As a result, creating the ideal educational and clinical training services is crucial and essentially entails customizing and changing each of its individual features. Each of them can be handled and viewed by a certain client independently. The quality of these services must be continuously assessed in order for the client to be completely happy, allowing for ongoing development of the nursing internship clinical training plan.

Nursing internship students are getting clinical experience and exposure in the clinical setting, which is essential for the development of clinical competence [8]. Nursing internship students must be prepared to work in a complicated setting with patients in diverse scenarios that need a higher degree of nursing clinical abilities [9], as well as, they must finish a 12-month internship program in various regions or specialized units of hospitals. Attitude development can be a direct personal experience or observation in which the individual begins to identify care in personal terms before moving on to professional identification, which takes more time than knowledge and abilities [10].

A thorough assessment of current internship training system and plan finds two key issues. Firstly, most published training programs are based on a general, ready-to-use curriculum that is not context-specific. This is especially important in the healthcare sector since there is a wide range of quality issues that differ depending on whether they exist in training, clinical, or technological procedures. Second, the content of the majority of internship training programs is defined by experienced trainers who predetermine the required information and skills that trainees will learn. Although appropriate for a vocational environment, this expert-driven training strategy does not perform well in a complicated health care setting when the bulk of personnel are highly educated professionals with distinct learning preferences [11–13]. Therefore, it is highly welcomed to notice and thoroughly analyze the clinical training quality assessment from the clients/nursing internship students using various quality improvement methodologies.

The Kano technique, according to Madzík et al., (2019) [7], can considerably help identify the qualities and needs of nursing students for efficient training. This approach is predicated on the idea that a given product's features, including those of its services, are multifaceted and have a range of effects on the degree of customer satisfaction. While some characteristics of the good or service tend to increase contentment, others help to increase discontent. As a result, knowing what the customer wants, it enables management to concentrate on creating the ideal features for the educational service. This method helps an organization manage its resources more thoroughly so that they can be moved as needed to best meet the needs of the client at any given time [7].

### Theoretical foundations of the study

**Fig. 1:**
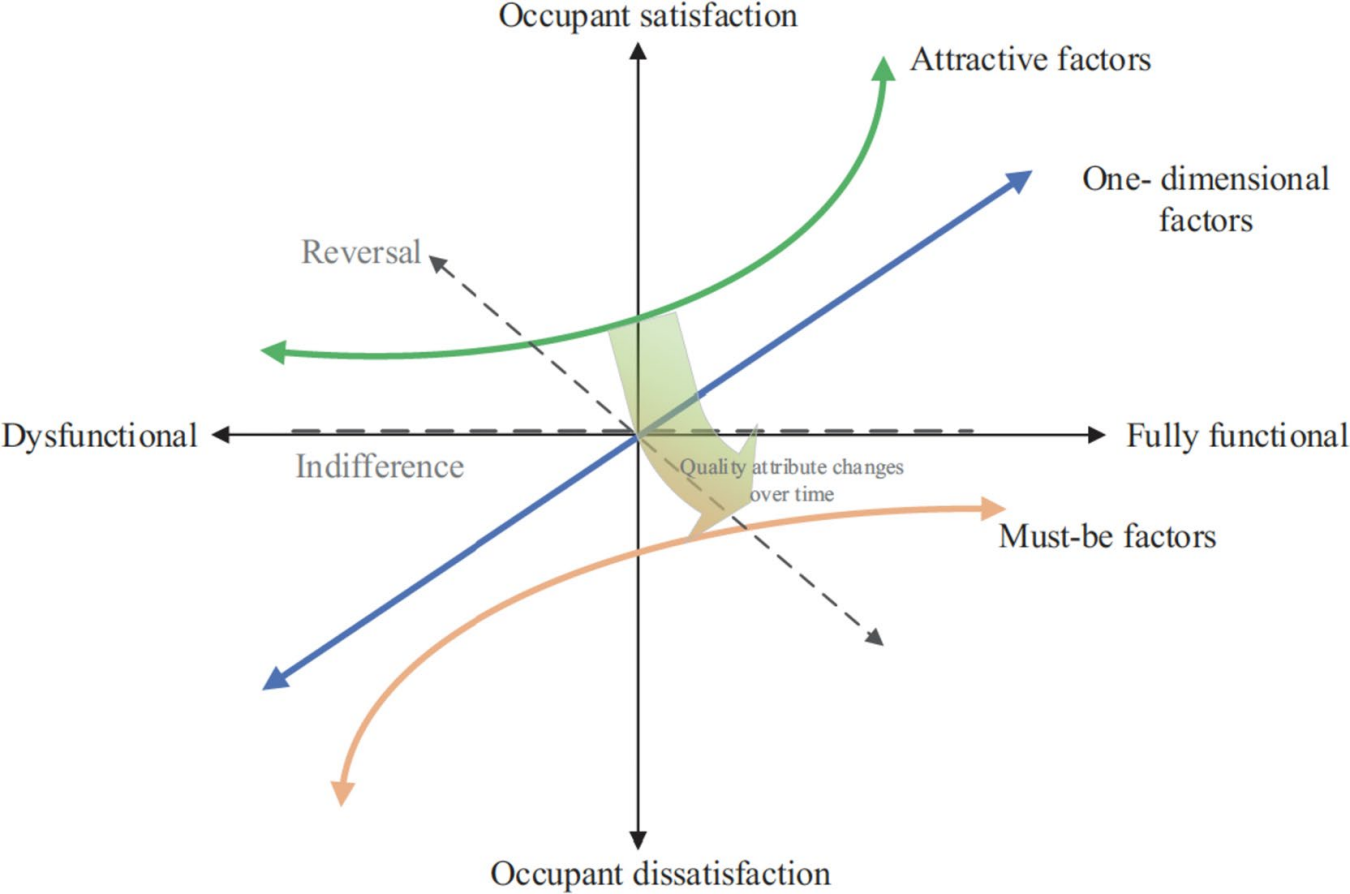
Kano’s satisfaction model and categories of attribute quality (adapted from Dace and Timma et al., 2020, Shahin, et al., 2013, Kano et al., 1984) [9–11]

The study is based on a major concept from the quality literature: the Kano model of customer satisfaction and its implications for categorizing customers' preference and requirements. To better understand the needs and expectations of internship nursing students, a variety of measures and methodologies have been developed. The Kano model is extremely useful for understanding the voice of the customer and influencing consumer satisfaction with the internship training. Kano proposed his hypothesis in response to the difficulty of a one-dimensional quality strategy in fully explaining consumer satisfaction [8–10]. According to the Kano model, customer satisfaction is not directly related to service capability levels. To put it another way, greater quality does not always translate into greater satisfaction with all aspects of a service. In some cases, minor improvements in service quality can significantly increase customer satisfaction; in others, large performance gains can only marginally increase customer satisfaction [10]. The Kano model describes the relationship between service performance/attributes and customer satisfaction. The horizontal axis represents a product's or service's level of performance or functionality, while the vertical axis represents the amount of customer satisfaction. Refer to Fig. 1.

All higher education institutions must seek appropriate solutions to societal requirements in this rapidly changing and increasingly unpredictable world [11]. Faced with numerous rapid changes and problems, as well as increased competition among firms to focus on their customers in order to improve their credibility. As a result, universities are attempting to describe the most important features for services that will help them meet client expectations and increase their popularity [13]. One difficult challenge that colleges face on their way to achieving this goal is how to conduct research on recognizing and realizing today's and tomorrow's requirements and expectations [14]. A review of the related literature reveals that quality improvement in higher education has been addressed in a variety of ways in the literature and numerous publications on the subject [15]. A significant portion of this research has focused on the examination of nursing internship students [16].

### Research Gap and significance statement

With little regard for internship nursing students’ training demands, the traditional approach to nursing internship training design in hospital settings is focused mostly on the trainers' experience. This strategy not only produces a wide variety of quality, application, and effectiveness in training system activities, but it also perceives nursing internship students as passive recipients of knowledge, abilities, and competencies. Even in surveys of consumer requirements, one-dimensional thinking is used, with the presumption that when customers' wants are met. The links between client expectations, satisfaction, and service quality are not properly explained by this one-dimensional approach [16]. Also, despite quality pioneers and accrediting bodies focus their recent philosophy in education toward inclusion of stakeholders and customers in all aspects of educational process to reach to better educational outcomes. Application of this philosophy is limited in nursing training especially the internship training of nursing students.

The current study's objective is to close the gap in nursing internship students' satisfaction with the internship training program that they receive to practice and link the theory with practice during the internship year by using the Kano analysis to explore the training quality attributes needed for improving the nursing internship training system from their perspectives. It also makes recommendations for implications in various nursing domains that will help to improve the quality of the nursing internship students' training and improve their satisfaction with the quality of their clinical practice. Additionally, there is a dearth of literature that focuses on the use of various quality models to enhance the efficient training system for nursing internship students but the literature was focusing on its application in higher education in general, an area that requires greater attention and study as nursing internship is a very rich training experience among the nursing internship students and it affect their clinical practice and satisfaction by the training experience. Although the Kano model has been employed relatively successfully in more technical areas, its promise in the field of education and internship clinical training has not been sufficiently recognized. But for a better understanding and prioritization of the demands and expectations of students toward the school, the Kano model is highly helpful. This article seeks to close this specific research hole.

### Aim

The study aims to explore the quality attributes required for optimizing the training system of nursing internship students using Kano model from the perspectives of nursing internship students.

### Research question

What are the quality attributes required for optimizing the training system of nursing internship students from their perspectives?

## Methods

### Design

A concurrent exploratory sequential triangulation mixed research design was used. The authors chose a mixed research method, an exploratory sequential design, to collect and analyze qualitative data before quantitative data. The triangulation process is chosen for generalizability, contextualization, and credibility. Qualitative research has a smaller sample size, while mixed methods offer large sample size external validity. Contextualization and credibility are enhanced by using qualitative data to illustrate quantitative findings. The convergence of qualitative and quantitative data strengthens the study's conclusion [17].

### Setting

In six government hospitals in Alexandria, Egypt, this study was carried out. Inpatient medical, surgical, and critical care units at these hospitals offer a variety of healthcare services. Due to the fact that the major teaching hospitals in the city were chosen as the study settings, each hospital had an average bed count that varied between 1,500 and 1,850. It had provided residents of Alexandria and the neighboring governorates with a wide variety of clinical acute therapies. Additionally, it had given nursing internship students the ability to learn and practice clinical skills, as well as a location for a variety of research initiatives.

In Egypt, a bachelor's degree in nursing is earned after four years of study, with an additional year of clinical internship. After completing the first four years of the academic and clinical learning process, which included theoretical foundations in nursing, nursing care management, and nursing professional growth, the student is acknowledged as a graduate. The freshly graduated nurse must then enroll in the internship program, which includes intensive training clinical courses with real patients. In an internship, the preceptorship model is used, in which a recently graduated nurse is reclassified as a preceptee under the supervision of a preceptor with clinical training. In order to give nursing graduates the chance to gain greater exposure to healthcare facilities and further develop their clinical competence, internship is a required component of nursing education in Egypt. The nursing interns will be exposed to and experience real patient care management in the clinical setting, which is essential for the development of clinical competence [9, 18].

### Study participants sampling

**Fig. 2:**
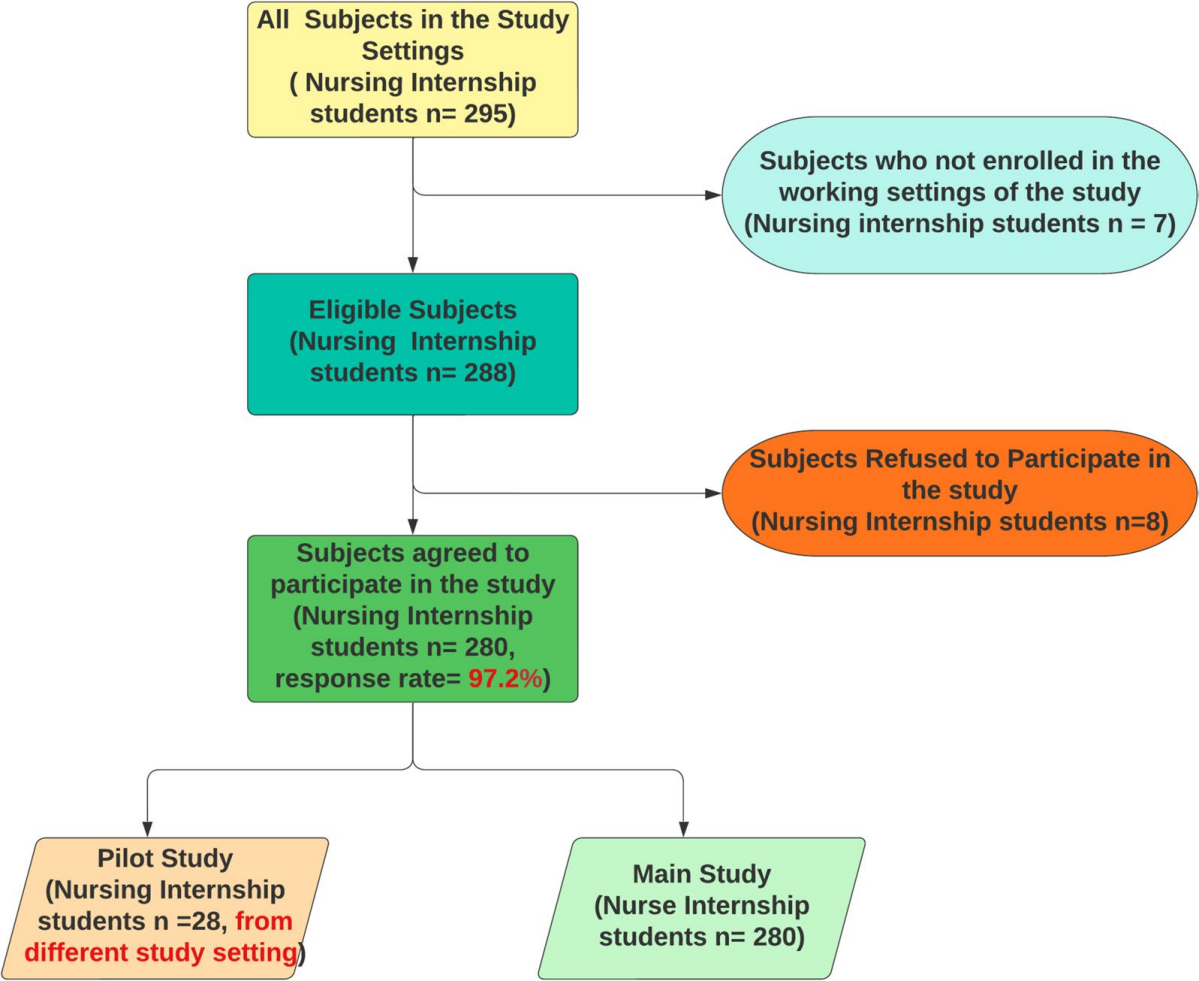
Sampling chart

A non-probability sampling technique was applied using convenience sampling of 295 nursing internship students were recruited from the different study settings in Egypt. Of them, 280 (97.2%) agreed to participate in the study and completed the interview and the self-administered questionnaire (Fig. 2). Inclusion Criteria were the nursing internship students who were working in their clinical settings for not less than two months. The authors included the total population as one of the main objectives of the mixed-research (Exploratory Sequential) methods that will help the authors in the generalizability of the study findings and conclusion.

### Study instruments

#### Tool 1: Nursing Internship students Semi-Structured Interview (NISSI)

It was created by Abdelhamid (2013) [19] and is designed to capture the qualitative aspects of a nursing internship training system based on nursing internship students' views, requirements, and preferences. It was made up of four questions. In the first two questions, nursing internship students were asked to recollect three positive and three negative experiences with previous training sessions. The third question asked participants to evaluate the present training system. The final question asked participants to describe the characteristics of their ideal nursing internship training program. All replies were classified and categorized and the thematic analysis were done based on the data collected. Finally, a comprehensive list of training preferences for nursing internship students was created.

#### Tool 2: Kano Style Questionnaire (KSQ)

The authors created it using the Kano model framework [9–11] to identify and rank nursing internship students' preferences for the nursing internship training system. This was done by grouping the preferences of the nursing internship students determined in the first step into discrete training characteristics. Two questions were asked for each staff choice; the first question assessed staff reactions to the existence of the expectation or requirement (functional form). The second question assessed the staff's reaction to the lack of the expectation or requirement (dysfunctional form). Both questions have five alternative answers: like, must be, neutral, live with, and dislike.

Combining nursing internship students' replies to both questions allows the Kano model to assign degrees of responses (from positive to negative) to one of six requirement categories: attractive (A), must-be (M), one-dimensional (O), indifferent (I), reversed (R), or questionable (Q). Each of these categories had a distinct influence on training system satisfaction (Table 1).

**Table 1:** Kano categorization matrix

**Nursing Internship students Requirements**		**Dysfunctional (absence) question**				
		**Like**	**Must be**	**neutral**	**Live with**	**Dislike**
**Functional (presence) question**	**Like**	**Q**	**A**	**A**	**A**	**O**
	**Must-be**	**R**	**I**	**I**	**I**	**M**
	**Neutral**	**R**	**I**	**I**	**I**	**M**
	**Live with**	**R**	**I**	**I**	**I**	**M**
	**Dislike **	**R**	**R**	**R**	**R**	**Q**

Furthermore, the authors created a socio-demographic and work-related form that contained questions about nursing internship students' gender, age, current training hospital, and internship experience.

#### Validity and reliability

The questionnaire was translated into Arabic and back into English. The questionnaire and the interview questions were then submitted to a panel of five experts (four Professors and one Lecturer from the Nursing Administration Department) who examined and assessed the content validity and offered feedback on the content, question types, and item clarity. Their comments were considered to ensure accuracy and to prevent possibly undermining the study. The questionnaire was tested for reliability by evaluating the items’ internal consistency using Cronbach’s alpha coefficient test. The questionnaire was tested for reliability by evaluating the items’ internal consistency using Cronbach’s alpha coefficient test. Out of the KSQ questionnaire, 6 items ( α = 0.945) were related to training policies and schedule, 7 items ( α = 0.933) were related to training content, 6 items ( α = 0.921) were related to training environment, 5 items ( α = 0.934) were related to internship students support, 3 items ( α = 0.991) were related to preceptorship, 4 items ( α = 0.897) were related to training incentives and motivation, 4 items ( α = 0.899) were related to autonomy, and the total questionnaire score (α = 0.924) was the average of the seven dimensions that was developed based on the semi-structured interview analysis. In addition, pilot research on 10% of nursing internship students was conducted to validate the tool's validity and reliability which resulted in no change.

## Data collection

The hospitals' administration gave formal permission for data gathering. The study questionnaire was sent to nursing internship students. Data was collected during a six-months period in 2022 (February to August). In this investigation, a two-phased strategy was used:

### Phase I: Utilizing voice of customers

In this phase, nursing internship students training preferences and needs were identified. Also, their opinions about quality attributes required to optimize their training are explored. This was accomplished through in-depth interviews with nursing internship students in the selected settings using tool (I). Results of interviews with nursing internship students were analyzed and a master list composed from 35 quality attributes was developed. The interview took around 40 min to acquire the necessary data.

### Phase II: Categorization and prioritization

This phase categorizes and ranks nursing internship students' indicated training choices and requirements. This was achieved by creating a Kano style questionnaire that addressed each of the 35 characteristics. The significance of each training feature was allocated using Kano analysis, in which nursing internship students’ answers were collected by frequency for each Kano category, and the final classification was based on the mode (the most frequent response). The questionnaire took between 10–15 min to complete.

### Ethical considerations

The Alexandria University Ethics Committee (N:12–2-2022) and the directors of the study settings granted formal approval to gather the necessary data. The confidentiality of the data were preserved. The nursing internship students signed consent to participate in the study prior to data collection was safeguarded. The participants were given the option to withdraw from the study at any time.

### Data analysis and management

The authors coded quantitative data and evaluated it with the IBM SPSS software program version 26.0. All reported p-values are two-tailed, and statistical significance was determined at the 0.05 level. Socio-demographic factors were described using frequency and percentages. To portray socio-demographic information and to display continuous variables, frequencies and percentages were used. For categorical variables, the Chi-square test was used to compare various groups. The Pearson coefficient was used to determine the relationship between two normally distributed quantitative variables.

O'Connor and Gipson's theme analysis technique was used to evaluate qualitative data (2003) [20]. The interviews were verbatim transcribed. The authors began with a familiarization stage in which the transcripts were read numerous times to gain a sense of completeness and a broad sense of the material before subjecting them to content analysis to discover emergent themes. The authors created preliminary codes by highlighting significant phrases and looking for common patterns within these codes. Then, to establish changing topics, categories were created by searching for similarities and variances across transcripts. Data analysis occurred concurrently with data collection; it began following the first interview and continued throughout the data gathering process.

To preserve data quality and rigor, all academic rigor criteria were evaluated, including credibility, transferability, dependability, and conformability. To increase the credibility of the findings, peer authors double-checked each other throughout the process to verify that the correct meaning was conveyed. Some interviewed nurse internship students checked the transcript to ensure that the interviewer accurately represented their points of view. No adjustments were proposed by the participants. Transferability was achieved by include a detailed explanation of the study techniques and data in the final research report to see whether the findings could be applied to a different demographic or study. A full methodology explanation was provided to assess dependability. Consistency checks were done by a peer researcher to create congruent viewpoints between two independent authors regarding the data's correctness, relevance, or significance to verify the dependability and conformability of the data analysis. The findings use verbatim quotations from participants to depict interview data.

A unique technique based on a one-dimensional chisquare method is suggested in this work. The highest and second highest categories are subjected to a one-dimensional chi-square test to see whether there is a statistically significant difference at the 90% confidence level. If the p value is less than 0.1, the difference in frequencies between the top two Kano categories is deemed substantial (i.e., a unimodal distribution), and the feature is allocated to the top Kano category. The difference in frequencies between the top two Kano categories is considered negligible if the *p* value is bigger than 0.1. (so essentially a bimodal or multimodal distribution).

## Results

The response rate is 97.2% (Fig. 2) after following up with all participants. Table 1 illustrates the nursing internship students’ demographic data. The nursing internship students’ mean age was 23.95 ± 1.44 years and 50.4% of the nursing internship students were females. As well as, 52.5% of the nursing internship students had an internship studentship training experience more than 6 months with a mean score 6.83 ± 2.38. Whereas the highest percentage (46.8%) of the nursing internship students were training at Alexandria Main University Hospital followed by 14.3% of them were training et al.-Hadara University hospital. The training choices expressed by nursing internship students were classified into seven broad categories based on the qualitative analysis of the nursing internship students responses on the interview, as shown in Table 2 that clarify the qualitative themes of the training attributes based on the nursing internship students’ preferences. It became clear that the nursing internship students' preferences reflected contextual difficulties related to the training system, policy, and environment. Nursing internship students indicated definite preferences for training regulations and schedules, training material, training atmosphere, intern support system, preceptorship, incentives and motivation, and autonomy, according to the findings. 280 nursing internship students completed the Kano questionnaire. The nursing internship students' responses were summarized by frequency for each Kano category, as shown in Table 3. Refer to Appendix (A). The final classification based on the mode (the most common answer) was also displayed using the Kano technique. Table 4 summarizes the results from Table 2 in terms of the frequency of nursing internship students' choices attributed to each of the Kano categories.

**Table 2:** Themes and attributes of nursing internship program based on nursing interns preferences

Themes	Required attributes
Training policies and schedule	• Flexible time schedule and block schedule • Training rounds include all specialties and sub specialties • Examination before and after each area to pass or fail • Control of time schedule by faculty • Equal chance for training in all areas for both male and female interns • Schedule the similar training units to be in order one by one in training plan
Training content	• Objectives and goals for each training area • Theoretical background revision before each area • Clinical booklet with log book for each area • Specialized team from faculty members to provide training in each area • Application of nursing process model and nursing care plan in the training year • Implementation of competency-based education and problem-based learning • Scheduled monthly focus group sessions that include representatives from interns and alumnae to discuss evolving problems in training
Training Environment	• Presence of lockers and dress room in clinical areas • Hot meals and snacks availability • Transportation support for remote areas and night shifts • Provision of one uniform and identification card for all interns • Detailed orientation to clinical settings before training • Provision of accessible accommodation or discount on the fees of accommodation for expatriates
Interns support system	• Available line of communication 24 h with faculty members responsible for internship affairs • Transparency in discussing complaints of interns • Available grievance procedure without fear of retribution • Presence of policy to prevent exploitation of interns in training settings • Application of mentorship program
Preceptorship	• Availability of preceptors at all times • Include preceptors from nursing alumnae in each unit in clinical settings • Chang the focus of preceptors from just attendance monitoring to on-job training and clinical teaching
Incentives and motivation	• Achievement certificate for each training area • Salary increases with bonus at regular times • Monthly monetary reward for the best performers in each area • Specified health insurance package for nursing interns
Autonomy	• Opportunity for membership in clinical settings' different committees • Formation of nursing internship advocacy committee • Adoption of peer review assessment and training strategy • Offering avenues for post graduates’ studies

**Table 3:** Aggregate responses to the Kano questionnaire (*n* = 280)

Nursing Interns’ Training Attributes	A		I		M		O		Q		R		Category
	**No**	**%**	**No**	**%**	**No**	**%**	**No**	**%**	**No**	**%**	**No**	**%**	
Policies and schedule	74	26.4	69	24.6	29	10.4	90	32.1	15	5.4	3	1.1	O
Training content	134	47.9	35	12.5	9	3.2	87	31.1	12	4.3	3	1.1	A
Training environment	33	11.8	32	11.4	89	31.8	111	39.6	15	5.4	0	0.0	O
Interns support	59	21.1	34	12.1	22	7.9	142	50.7	14	5.0	9	3.2	O
Preceptorship	84	30.0	49	17.5	14	5.0	111	39.6	17	6.1	5	1.8	O
Incentives and motivation	58	20.7	27	9.6	15	5.4	159	56.8	18	6.4	3	1.1	O
Autonomy	138	49.3	68	24.3	6	2.1	56	20.0	12	4.3	0	0.0	A

*O* One–dimensional, *Q* Questionable, *I* Indifferent, *M* Must–be, *A* Attractive, *R* Reversal

**Table 4:** Distribution of the nursing interns according to categories resulting of Kano questionnaire (*n* = 280)

**Categories**	No	%
**Attractive**	83	29.6
**Indifferent**	45	16.1
**Must- be**	29	10.4
**One – dimensional**	105	37.5
**Questionable**	15	5.4
**Reversal**	3	1.1
**Total**	280	100%

The nursing internship students' quality attributes were classified as follows based on the mixed method analysis of their training preferences following the Kano-model categorization matrix:

### Attractive quality attributes (A)

The following features were rated as appealing by 29.6% of respondents: training content theoretical basis, clinical booklet and log book of the training area, nursing process application and nursing care plan in the training year, involvement of a specialized team from faculty members to provide the clinical training, application of competency-based and problem-based learning in internship students training, focus group sessions that include representatives from internship students and alumnae to discuss evolving problems in training monthly, accessible accommodation or discount on the fees of accommodation for expatriates, availability of communication 24 h with faculty members responsible for internship affairs, availability of mentorship program in the training system of nursing internship students, availability of preceptors from nursing alumnae in each unit in clinical settings, preceptors focus on-job training and clinical teaching, achievement certificate to be awarded after each training area, monthly monetary reward for the best performers in each area in the training year, internship studentship program offers opportunity for nursing internship students to participate in the different committees of the training settings, training system of nursing internship program adopts a peer review assessment strategy during the training year, training system of nursing internship program is based on exam before and after each area as a requirement to pass or fail in this area, time schedule (Roster) of nursing internship students is controlled by faculty not by the training settings, training system of nursing internship program is based on scheduling the similar training units to be in order one by one in training plan, and the training system of nursing internship program offers avenues for nursing internship students to continue their postgraduate studies. (Refer to Appendix A- Table SUP 1–6).

### Indifferent quality attributes (I)

16.1% of features were categorized as indifferent and these were: the training system of nursing internship program was based on flexible time schedule (Roster) and block schedule, and require rounds/ rotations in all specialties and sub specialties. (Refer to Appendix A- Table SUP 1–6).

### Must-be quality attributes (M)

The following attributes were identified as must-haves by 10.4% of respondents: the training system of nursing internship program includes objectives and goals for each training area, there are lockers and dress rooms available and specified for nursing internship students during the training year, there is a transportation support for remote areas and night shifts available daily for nursing internship students during the training year, the training system of nursing internship program starts with detailed orientation to clinical settings before each training area, the training system of nursing internship program includes available grievance procedure (nursing internship students without fear of retribution, complaints of nursing internship students are discussed with transparency during the training year, and there is a valued salary with regular bonus increase for nursing internship students during the training year. (Refer to Appendix A- Table SUP 1–6).

### One-dimensional quality attributes (O)

The following were 37.5% of the traits: there are hot meals and snacks available for nursing internship students every shift during the training year, and there is a policy to prevent exploitation of nursing internship students in the training settings. (Refer to Appendix A- Table SUP 1–6).

### Reverse quality attributes (R)

1.1% of characteristics were classified as reversals, and they were: the training system of nursing internship studentship program is based on equal chance for training in all areas for both male and female internship students, and there is specified health insurance package available for nursing internship students during the training year. (Refer to Appendix A- Table SUP 1–6).

### Questionable quality attributes (Q)

5.4% of features were categorized as a questionable and that were: there are one uniform and identification cards available and specified for nursing internship students during the training year, there is a nursing internship advocacy committee that advocate for the rights of nursing internship students during the training year, and preceptors are available during the three shifts for education and mentoring. (Refer to Appendix A- Table SUP 1–6).

In this study, a rule states that a "must-be" category takes precedence over a "one-dimensional" category, which takes priority over a "attractive" assignment, which takes priority over a "indifferent" assignment, which may be represented as M > 0 > A > I [8–10]. This is a cautious approach to ensure that aspects that may have a negative influence on nursing intern satisfaction are addressed before those that contribute to satisfaction. Table 5 shows the Chi-Square test results for each of the seven training characteristics. The p values indicated a high significant importance for nursing internship students' satisfaction with the internship studentship training across the training content, internship students' support, preceptorship, incentives and motivation, and autonomy, with *p* values = 0.002, 0.001, 0.001, and 0.001, respectively. The strategy for deploying quality functions was based on recognizing customer requirements and preferences and providing a priority level to these preferences. Using the Kano model, nursing internship students internship training quality attributes and preferences were identified and classified into seven themes with variable effects on satisfaction. (Refer to Table 5).

**Table 5:** Kano categories of the training attributes based on statistical significance (*n* = 280)

Nursing Interns’ Training Attributes	Most Frequency	Second Most Frequency	p	Significant difference	Final category
Policies and schedule	O	A	0.212	No	O
Training content	A	O	0.002*	Yes	A
Training environment	O	M	0.120	No	M
Interns support	O	A	< 0.001*	Yes	O
Preceptorship	O	A	0.053*	Yes	O
Incentives and motivation	O	A	< 0.001*	Yes	O
Autonomy	A	I	< 0.001*	Yes	A

*O* One–dimensional, *Q* Questionable, *I* Indifferent, *M* Must–be, *A* Attractive, *R* Reversal *p* p for Chi square test

^*^: Statistically significant at *p* ≤ 0.1

^**^: Based on M > O > A > I rule

In final Category

M take priority over O

O take priority over A

A take priority over I

It was also feasible to generate a numerical significance rating by adding the frequencies in each of the Kano categories using the following equations [8–10]:

1 $$\mathrm{Satisfaction}\;\mathrm{Index}=\frac{\mathrm A+\mathrm O}{\mathrm A+\mathrm O+\mathrm M+\mathrm I}$$

2 $$\mathrm{Dissatisfaction}\;\mathrm{Index}=\frac{\mathrm M+\mathrm O}{\mathrm A+\mathrm O+\mathrm M+\mathrm I}$$

These formulas provide a satisfaction index ranging from 0 to 1 and a dissatisfaction index ranging from 0 to -1. The relevance rating in the quality function deployment matrix would be based on the greater of these two indices' absolute values transformed into a percentage. This method to priority rating computations would prioritize boosting satisfaction above limiting unhappiness, which would be consistent with the cautious approach used in Kano category assignments. Table 6 showed the outcomes of applying the aforesaid strategy to employee preferences.

**Table 6:** Importance rating of the training attributes (*n* = 280)

Q	Training attributes	A				I		M	O	Q	R	Satisfaction index	Dissatisfaction index	Importance rating	Rank
A															
1	**Policies and Schedule:** How would you feel if the training system of nursing internship program was based on flexible time schedule(Roster) and block schedule	44				100		70	32	26	8	0.31	-0.41	41.5%	**34**
2	How would you feel if the training system of nursing internship program require rounds/ rotations in all specialties and sub specialties?	77				111		47	32	8	5	0.41	-0.30	40.8%	**35**
3	How would you feel if the training system of nursing internship program is based on exam before and after each area as a requirement to pass or fail in this area?	120				51		27	36	24	22	0.67	-0.27	66.7%	**17**
4	How would you feel if time schedule (Roster) of nursing interns is controlled by faculty not by the training settings?	113				66		17	26	12	46	0.63	-0.19	62.6%	**25**
5	How would you feel if the training system of nursing internship program is based on equal chance for training in all areas for both male and female interns?	29				70		135	28	12	6	0.22	-0.62	62.2%	**27**
6	How would you feel if the training system of nursing internship program is based on scheduling the similar training units to be in order one by one in training plan?	118				60		29	49	15	9	0.65	-0.30	65.2%	**22**
7	**Training content:** How would you feel if the training system of nursing internship program includes objectives and goals for each training area?	27				40		159	21	24	9	0.19	-0.73	72.9%	**7**
8	How would you feel if the training system of nursing internship program includes theoretical background revision before each area?	128				26		33	57	15	21	0.76	-0.37	75.8%	**3**
9	How would you feel if the training system of nursing internship program includes clinical booklet with log book for each area?	119				60		21	56	15	9	0.68	-0.30	68.4%	**14**
10	How would you feel if the training system of nursing internship program involves application of nursing process model and nursing care plan in the training year?	158				47		19	27	18	11	0.74	-0.18	73.7%	**6**
11	How would you feel if the training system of nursing internship program includes a specialized team from faculty members to provide training in each clinical area?	135				62		29	40	5	9	0.66	-0.26	65.8%	**21**
12	How would you feel if the training system of nursing internship program applies the competency based education and problem based learning in the training of interns?	137				75		14	40	11	3	0.67	-0.20	66.5%	**18**
13	How would you feel if the training system of nursing internship program includes scheduled monthly focus group sessions that include representatives from interns and alumnae to discuss evolving problems in training?	140				90		6	24	9	11	0.63	-0.12	63.1%	**24**
B															
14	**Training environment:** How would you feel if there are lockers and dress rooms available and specified for nursing interns during the training year?	34			54			136	38	12	6	0.27	-0.66	66.4%	**19**
15	How would you feel if there are hot meals and snacks available for nursing interns every shift during the training year?	30			52			152	28	12	6	0.22	-0.69	68.7%	**13**
16	How would you feel if there is a transportation support for remote areas and night shifts available daily for nursing interns during the training year?	81			48			83	53	12	3	0.51	-0.51	51.3%	**31**
17	How would you feel if there are one uniform and identification cards available and specified for nursing interns during the training year?	44			75			92	46	11	12	0.35	-0.54	53.7%	**30**
18	How would you feel if the training system of nursing internship program starts with detailed orientation to clinical settings before each training area?	30			65			132	35	15	3	0.25	-0.64	63.7%	**23**
19	How would you feel if nursing internship program includes accessible accommodation or discount on the fees of %accommodation for expatriates?	114			41			39	68	12	6	0.69	-0.41	69.5%	**11**
20	**Interns support:** How would you feel if the training system of nursing internship program includes available line of communication 24 h with faculty members responsible for internship affairs?	156			36			15	52	9	12	0.80	-0.26	**80.3%**	**1**
21	How would you feel if the training system of nursing internship program includes available grievance procedure (nursing interns without fear of retribution?	47			48			127	30	12	16	0.31	-0.62	62.3%	**26**
22	How would you feel if there is a policy to prevent exploitation of nursing interns in the training settings?	21			53			150	27	11	18	0.19	-0.71	70.5%	**8**
23	How would you feel if complaints of nursing interns are discussed with transparency during the training year	27			37			157	35	12	12	0.24	-0.75	75.0%	**4**
24	How would you feel if the mentorship program is applied in the training system of nursing interns?	131			72			12	45	15	5	0.68	-0.22	67.7%	**15**
25	**Preceptorship:** How would you feel if the training system of nursing internship program includes preceptors from nursing alumnae in each unit in clinical settings?	120			55			21	59	17	8	0.70	-0.31	70.2%	**10**
26	How would you feel if preceptors are available during the three shifts for education and mentoring?	105			60			56	39	9	11	0.55	-0.37	55.4%	**29**
27	How would you feel if preceptors focus changed from just attendance monitoring to on-job training and clinical teaching?	99			94			36	29	7	15	0.50	-0.25	49.6%	**33**
C															
28	**Incentives and motivation:** How would you feel if the training system of nursing internship program includes achievement certificate to be awarded after each training area?		134	38			14		62	15	17	0.79	-0.31	79.0%	**2**
29	How would you feel if there is specified health insurance package available for nursing interns during the training year?		46	79			99		29	9	18	0.30	-0.51	50.6%	**32**
30	How would you feel if the training system of nursing internship program includes monthly monetary reward for the best performers in each area?		113	56			25		66	17	3	0.69	-0.35	68.8%	**12**
31	How would you feel if there is a valued salary with regular bonus increase for nursing interns during the training year?		37	60			123		33	18	9	0.28	-0.62	61.7%	**28**
32	**Autonomy:** How would you feel if the training system of nursing internship program offers opportunity for nursing interns to participate in the different committees of the training settings?		134	77			9		40	9	11	0.67	-0.19	66.9%	**16**
33	How would you feel if the training system of nursing internship program adopts a peer review assessment strategy during the training year?		145	69			18		27	12	9	0.66	-0.17	66.4%	**20**
34	How would you feel if there is a nursing internship advocacy committee that advocate for the rights of nursing interns during the training year?		166	51			15		30	15	3	0.75	-0.17	74.8%	**5**
35	How would you feel if the training system of nursing internship program offers avenues for nursing interns to continue their postgraduate studies?		150	61			17		35	14	3	0.70	-0.20	70.3%	**9**

*O* One–dimensional, *Q* Questionable, *I* Indifferent, *M* Must–be, *A* Attractive, *R* Reversal

This method offered two levels of classification for each training choice, as can be shown. The Kano category came first, followed by a numerical significance rating. For example, having a training system of nursing internship program that includes available line of communication 24 h with faculty members responsible for internship affairs (importance rate 80.3%) and having training system of nursing internship program includes achievement certificate to be awarded after each training area (importance rate 79%), both preferences were classified as "Attractive," and the priority rating for the training system of nursing internship program that includes theoretical background revision before each area (75.8%) was much higher than complaints of nursing internship students are discussed with transparency during the training year (75%). Furthermore, all of these characteristics were much higher than the nursing internship advocacy committee (74.8%), which advocates for the rights of nursing internship students during the training year. (Refer to Table 6a, b and c.

## Discussion

The current study provides the fundamental characteristics required to develop a high-quality nursing internship program using the voice of customers (VOC) approach. Furthermore, the current study's findings revealed 35 quality attributes required to optimize the nursing internship program's training system. Meanwhile, incorporating these characteristics into the nursing internship program is critical to increasing nursing internship students’ satisfaction and competency. Kano analysis was used to estimate the degree of importance of each attribute in relation to the nursing internship after the Kano model was used to classify the quality attributes obtained.

Kano analysis revealed that 22 out of 35 attributes are appealing to nursing internship students, and fulfillment of these attributes leads to an exponential increase in nursing intern satisfaction. Furthermore, 11 of the 35 attributes are classified as mandatory. Those characteristics, if absent or unmet, result in extreme intern dissatisfaction and a lack of intern interest in mastering the core competencies of the nursing internship program. Furthermore, two attributes are indifferent to nursing internship students, and fulfillment of those attributes has no effect on nursing internship satisfaction. The analysis of nursing internship students’ responses yields seven themes that shape the critical pillars of the nursing internship program. It includes training policies and schedules, training content, training environment, nursing intern support, preceptorship, incentives and motivation, and nursing internship students’ autonomy. Each theme embrace number of quality attributes that required to optimize the current nursing internship program.

Training policies and schedule is the backbone of nursing internship programs however; nursing internship students in the current study expressed many drawbacks in this area. High discrimination among internship students, unequal chance for training, and poor ordering of related modules are the most reported experiences. These experiences may be due to refusal of some Alexandria university hospitals to provide training to male internship students especially in the areas of obstetrics & gynecology and pediatrics. These experiences were reported in other studies as Abdelhamid (2013) [18]; Jamshidi et al. (2016) [19]; Uche et al., (2017) [21]; Abd elrahman, Eid& Safaan(2021) [22]. In contrary, the studies of Maertz et al., (2014) [23]; Stack and Fede (2017) [24] found high satisfaction among internship students in relation to training policies with no discrimination reported.

To get out of all these shortcomings, nursing internship students expressed six preferences that need to be included in the internship program. They reported that the training system of nursing internship studentship program must be based on equal chance for training in all areas for both male and female internship students. At the same time, controlling of time schedule by educational institution, scheduling the similar training units to be in order one by one in training plan, conducting exam before and after each area as a requirement to pass or fail in this area are attractive attributes expressed by nursing internship students. On the other hand, flexible and block scheduling of training, including all specialties and sub specialties in training rounds were categorized as indifferent attributes by the majority of nursing internship students. Training content is a major theme that had many weaknesses in the current study. The highest areas that internship students shed the light are poor application of log book, objectives of each module not clear, nursing process not applied in clinical settings, and lack of emphasis on theoretical knowledge required for each module. This result may be due to limited focus on nursing process during undergraduate period. These drawbacks are also expressed in the studies of Abdelhamid (2013) [18]; AlThiga, et al. (2017) [5]; Githui & Wambui (2019) [25]; Alnajjar et al. (2019) [26]. On the other hand, the studies of Ghazy and Shahat (2021) [27]; Tindowen et al., (2019) [28]; and Zehr and Korte (2019) [29] found nursing internship students satisfied with the content of internship program in which nursing process is applied in the different modules.

Furthermore, providing sound training content is big challenge for internship program planners however, nursing internship students in the current study put the foundations in which training content articulated such as clear objectives of training, application of nursing process model, implementation of competency-based learning, and conducting theoretical background revision before each training area. Moreover, using of log book for each training area, availability of faculty members to provide training in each area, and conducting monthly focus group sessions to discuss evolving problems in training are also other pillars of sound training content. It is evident that the training environment ignores the physical and psychosocial needs of nursing internship students in the current study. This is reflected in the nature of attributes reported by nursing internship students since these attributes articulated around the basic needs that must be fulfilled in the training environment. These attributes include provision of lockers, dress room in training areas, accessible accommodation, hot meals and snacks, one uniform, and identification card for all internship students. Also, detailed orientation to clinical settings before training is another issue that received consensus among nursing internship students. This outcome could be attributed to a lack of resources in Alexandria university hospitals. The current study's findings are consistent with those of Githui and Wambui (2019) [25], AlThiga et al., (2017) [5], and Safan and Ebrahim (2018) [30], who discovered significant flaws in the training environment for nursing internship students. These findings contradict the findings of Wei et al., (2021) [31], Mauhay (2016) [32], and Ghazy et al., (2021) [27], who discovered that the facilities and resources required for internship nursing students’ training are available.

Concerning preceptorship system in the current study, it faced with many weaknesses which represented by shortcomings and remedies reported by nursing internship students. This could be due to the limited number of preceptors available for intern training, as well as the scarcity of preceptor continuing education programs. Meanwhile, nursing internship students highlighted many features to improve the current preceptorship system. Availability of preceptors at all times, and changing the focus of preceptors from just attendance monitoring to on-job training and clinical teaching are the most frequently and reported preferences. The findings of the present study are parallel with Ghazy et al. (2021) [27], Ahanchian et al., (2017) [33] who found many problematic areas in the preceptorship system of nursing internship students. Also, nursing internship students in the current study prefers to assign the supervisory role of preceptors to the experienced nursing staff. This is go with opinion of Al-Mamari et al., (2015) [34] who highlighted that when the highly experienced nurses act as preceptors, it give strong ambition to nursing internship students to proceed in their careers. In contrast, Alkaya and Terzi's (2021) [35] study found that internship students were more satisfied with the role of preceptors.

It is a worthy to note that words of nursing internship students in the current study reflect feeling of insecurity with poor support from faculty and clinical settings. This could be due to a shortage of nurses in Alexandria university hospitals, as well as a workload distribution bias toward internship students. These results are in the same line with Safan & Ebrahim (2018) [30], Makhlof & El-Saman (2017) [36], and Ahmadi et al., (2021) [37] who found serious obstacles that hinder the satisfaction of nursing internship students such lack of support system, interaction difficulties with staff, ambiguity in the evaluation system, and obscurity in identity. In contrast, Alharbi and Alhosis (2019) [1] discovered that internship students received a lot of help from educational and clinical settings during their training. Fortunately, nursing internship students in the current study suggested many proposals to cultivate the security and support feelings. These proposals include establishing available line of communication 24 h with faculty members responsible for internship affairs, setting grievance procedure without fear of retribution, and developing a policy to prevent exploitation of internship students in training settings. Moreover, application of mentorship program and transparency in addressing complaints of internship students are critical proposals highlighted by the majority of nursing internship students.

The autonomy of nursing internship students is a critical area in the current study because the majority of nursing internship students feel indecisive. This finding reflects the general situation of nursing in Egypt, which has limited space for shared decision making and job control. It is not supersizing result as perceived low levels of autonomy is a common pattern in many studies such as Thabet et al., (2020) [38]; Lawal et al., (2016) [39]; Sürücü et al., (2021) [40]; Turan et al., (2017) [41]. These findings contradict those of Al Najjar et al., (2019) [26], who discovered increased autonomy among nursing internship students. Nursing internship students in the current study put much emphasis on the attributes required for autonomous trainees. Participating of nursing internship students in different committees of training settings and formation of nursing internship advocacy committee are two modalities expressed by nursing internship students and required for maximum autonomy. Also, adoption of peer review assessment and training strategy as well as offering avenues for nursing internship students for post graduates studies are other positive ways for maintaining autonomy which reflected in the thematic analysis of nursing internship students' responses.

Motivation is an imperative theme required for effective training however; responses of nurses' internship students in the current study reflect lack of interest and motivation with poor commitment to their training program and training settings. This finding is attributed to the intern's low monetary reward as well as the lack of recognition of internship students' effortsand contribution. This is a common finding in different studies like Duprez et al., (2021) [42]; De Los Santos et al., [43]; and Ghazy et al., (2021) [27]. Conversely, the study of Wang et al., (2021) [44] discovered a high level of motivation among internship students in their study. To sustain motivation in training, nursing internship students in the current study give the remedies to achieve this aim such as salary increase with bonus at regular times, and specifying monthly monetary reward for the best performers in each area. Meanwhile, awarding achievement certificate after each training area as well as specifying health insurance package for nursing internship students are two motives that expressed by the highest percentage of nursing internship students.

## Conclusion

The aim of the current study was to identify the training quality attributes required to optimize the training system's quality for nursing internship programs using the Kano model. The difficulties of the nursing students' internship training were investigated, as well as prospective solutions for its quality enhancement, using the voice of the customer technique. 35 fundamental elements were identified by the Kano-model analysis as being necessary for high-quality nursing internship training in order to increase the students' satisfaction with their clinical experience and education. The 22 quality attributes that were classified as "attractive," 11 that were classified as "must be," and two that were classified as neutral were the ones that should be carefully considered while planning and modifying the nursing internship training system.

### Implications of the study

#### Implications for nursing management

The training program of nursing internship students shapes their future career so it is necessity to build sound program. It is implied from the current study that building effective nursing internship is articulated around seven critical pillars namely; training policies and schedule, training content, training environment, preceptorship, internship students support system, incentives, and autonomy avenues. Nurse managers in clinical settings should put much emphasis on theses pillars to ensure that nursing internship students are mastering the skills of competent alumni. It is imperative to provide conducive training environment that fulfill at least the basic needs of internship students to maintain passion for learning as well as commitment of internship students to nursing profession. Also, incorporating internship students support system with motivation strategies are helpful tools to maintain exemplary performance of internship students during the training period.

#### Implications for nursing education

It is inferred from the current study that nurse educators should use voice of customers approach to upgrade the internship program of nursing internship students. Nurse educators should consider the fact that not all features of training system of internship take the same importance to nursing internship students so it is vital to identify which features take the priority than others. Also, conducting theoretical background revision before each training module is helpful strategy to link theory with practice. Moreover, the internship program must be based on clear intended learning outcomes that should be updated at regular intervals. It is the responsibility of nurse educators to match the intended learning outcomes of the training with internship students' needs and expectations through needs assessment tools. Finally, educational bodies should maintain open direct channels of communication with internship students' training settings for rapid coordination and feedback.

#### Implications for nursing research

To establish generalizability of results, the study should be replicated on a larger probability sample. More research is needed to determine the effect of adopting the quality aspects of the internship program discovered in the current study on the performance of nursing internship students.

#### Research strengths and limitations

This is one of the first studies to use a mixed-methods study (Exploratory sequential triangulation design) to investigate the quality attributes required for optimizing the training system of nursing internship students by utilizing and applying the Kano model among nursing internship students in Egypt from their perspectives. It provides a comprehensive picture of all quality attributes affecting the nursing internship training system. Furthermore, the thematic framework presented in this paper based on qualitative data may serve as a snapshot for future improvement and evaluation strategies by policymakers, health managers, and the nursing syndicate. This study may indeed, however, have some limitations that must be acknowledged. The sample drawn from a single large university hospital is not representative of all Egyptian nursing internship students. This will limit the findings' generalizability among nursing internship students. Furthermore, because the exploratory sequential research design was used, the identified variables may not be comprehensive variables. Because the sample only included nursing internship students from Alexandria University, it is recommended that future studies include nursing internship students from other universities.

### Supplementary Information

**Additional file 1:** **Table S1.** Distribution of the studied cases according to training policies and schedule Theme (*n* = 280). **Table S2.** Distribution of the studied cases according to training content Theme (*n* = 280). **Table S3.** Distribution of the studied cases according to training environment Theme (*n* = 280). **Table S4.** Distribution of the studied cases according to interns’ support Theme (*n* = 280). **Table S5.** Distribution of the studied cases according to preceptorship Theme (*n* = 280). **Table 6.** Distribution of the studied cases according to incentives and motivation Theme (*n* = 280). **Table S7.** Distribution of the studied cases according to autonomy Theme (*n* = 280).

## Data Availability

The data and materials of the current study are not publicly available due to confidentiality reason but are available from the corresponding author on reasonable request.
