# Supplementary material for: Application of Kano model for optimizing the training system among nursing internship students: a mixed-method Egyptian study
Source: BMC Nurs. 2023 Sep 14;22:316. doi: 10.1186/s12912-023-01485-5 (PMC10500916; doi:10.1186/s12912-023-01485-5)
Supplement: Supplementary file 1 — Additional file 1: Table S1. Distribution of the studied cases according to training policies and schedule Theme (n = 280). Table S2. Distribution of the studied cases according to training content Theme (n = 280). Table S3. Distribution of the studied cases according to training environment Theme (n = 280). Table S4. Distribution of the studied cases according to interns’ support Theme (n = 280). Table S5. Distribution of the studied cases according to preceptorship Theme (n = 280). Table 6. Distribution of the studied cases according to incentives and motivation Theme (n = 280). Table S7. Distribution of the studied cases according to autonomy Theme (n = 280). [file 12912_2023_1485_MOESM1_ESM.docx]

**Appendix (A)**

**Table (SUP 1): Distribution of the studied cases according to training policies and schedule Theme (n = 280)**

| **Q** | **Training policies and schedule** | **A** | | **I** | | **M** | | **O** | | **Q** | | **R** | | **Category** |
| --- | --- | --- | --- | --- | --- | --- | --- | --- | --- | --- | --- | --- | --- | --- |
|  |  | **No.** | **%** | **No.** | **%** | **No.** | **%** | **No.** | **%** | **No.** | **%** | **No.** | **%** |  |
| 1 | How would you feel if the training system of nursing internship program was based on flexible time schedule(Roster) and block schedule | 44 | 15.7 | 100 | 35.7 | 70 | 25.0 | 32 | 11.4 | 26 | 9.3 | 8 | 2.9 | **I** |
| 2 | How would you feel if the training system of nursing internship program require rounds/ rotations in all specialties and sub specialties? | 77 | 27.5 | 111 | 39.6 | 47 | 16.8 | 32 | 11.4 | 8 | 2.9 | 5 | 1.8 | **I** |
| 3 | How would you feel if the training system of nursing internship program is based on exam before and after each area as a requirement to pass or fail in this area? | 120 | 42.9 | 51 | 18.2 | 27 | 9.6 | 36 | 12.9 | 24 | 8.6 | 22 | 7.9 | **A** |
| 4 | How would you feel if time schedule (Roster) of nursing interns is controlled by faculty not by the training settings? | 113 | 40.4 | 66 | 23.6 | 17 | 6.1 | 26 | 9.3 | 12 | 4.3 | 46 | 16.4 | **A** |
| 5 | How would you feel if the training system of nursing internship program is based on equal chance for training in all areas for both male and female interns? | 29 | 10.4 | 70 | 25.0 | 135 | 48.2 | 28 | 10.0 | 12 | 4.3 | 6 | 2.1 | **R** |
| 6 | How would you feel if the training system of nursing internship program is based on scheduling the similar training units to be in order one by one in training plan? | 118 | 42.1 | 60 | 21.4 | 29 | 10.4 | 49 | 17.5 | 15 | 5.4 | 9 | 3.2 | **A** |
|  | **Total Policies and schedule** | **74** | **26.4** | **69** | **24.6** | **29** | **10.4** | **90** | **32.1** | **15** | **5.4** | **3** | **1.1** | **O** |

**Note. O: One–dimensional, Q: Questionable, I: Indifferent, M: Must–be, A: Attractive, R: Reversal**

**Table (SUP 2): Distribution of the studied cases according to training content Theme (n = 280)**

| **Q** | **Training content** | **A** | | **I** | | **M** | | **O** | | **Q** | | **R** | | **Category** |
| --- | --- | --- | --- | --- | --- | --- | --- | --- | --- | --- | --- | --- | --- | --- |
|  |  | **No.** | **%** | **No.** | **%** | **No.** | **%** | **No.** | **%** | **No.** | **%** | **No.** | **%** |  |
| 7 | How would you feel if the training system of nursing internship program includes objectives and goals for each training area? | 27 | 9.6 | 40 | 14.3 | 159 | 56.8 | 21 | 7.5 | 24 | 8.6 | 9 | 3.2 | **M** |
| 8 | How would you feel if the training system of nursing internship program includes theoretical background revision before each area? | 128 | 45.7 | 26 | 9.3 | 33 | 11.8 | 57 | 20.4 | 15 | 5.4 | 21 | 7.5 | **A** |
| 9 | How would you feel if the training system of nursing internship program includes clinical booklet with log book for each area? | 119 | 42.5 | 60 | 21.4 | 21 | 7.5 | 56 | 20.0 | 15 | 5.4 | 9 | 3.2 | **A** |
| 10 | How would you feel if the training system of nursing internship program involves application of nursing process model and nursing care plan in the training year? | 158 | 56.4 | 47 | 16.8 | 19 | 6.8 | 27 | 9.6 | 18 | 6.4 | 11 | 3.9 | **A** |
| 11 | How would you feel if the training system of nursing internship program includes a specialized team from faculty members to provide training in each clinical area? | 135 | 48.2 | 62 | 22.1 | 29 | 10.4 | 40 | 14.3 | 5 | 1.8 | 9 | 3.2 | **A** |
| 12 | How would you feel if the training system of nursing internship program applies the competency based education and problem based learning in the training of interns? | 137 | 48.9 | 75 | 26.8 | 14 | 5.0 | 40 | 14.3 | 11 | 3.9 | 3 | 1.1 | **A** |
| 13 | How would you feel if the training system of nursing internship program includes scheduled monthly focus group sessions that include representatives from interns and alumnae to discuss evolving problems in training? | 140 | 50.0 | 90 | 32.1 | 6 | 2.1 | 24 | 8.6 | 9 | 3.2 | 11 | 3.9 | **A** |
|  | **Total training content** | **134** | **47.9** | **35** | **12.5** | **9** | **3.2** | **87** | **31.1** | **12** | **4.3** | **3** | **1.1** | **A** |

**Note. O: One–dimensional, Q: Questionable, I: Indifferent, M: Must–be, A: Attractive, R: Reversal**

**Table (SUP 3): Distribution of the studied cases according to training environment Theme (n = 280)**

| **Q** | **Training Environment** | **A** | | **I** | | **M** | | **O** | | **Q** | | **R** | | **Category** |
| --- | --- | --- | --- | --- | --- | --- | --- | --- | --- | --- | --- | --- | --- | --- |
|  |  | **No.** | **%** | **No.** | **%** | **No.** | **%** | **No.** | **%** | **No.** | **%** | **No.** | **%** |  |
| 14 | How would you feel if there are lockers and dress rooms available and specified for nursing interns during the training year? | 34 | 12.1 | 54 | 19.3 | 136 | 48.6 | 38 | 13.6 | 12 | 4.3 | 6 | 2.1 | **M** |
| 15 | How would you feel if there are hot meals and snacks available for nursing interns every shift during the training year? | 30 | 10.7 | 52 | 18.6 | 152 | 54.3 | 28 | 10.0 | 12 | 4.3 | 6 | 2.1 | **O** |
| 16 | How would you feel if there is a transportation support for remote areas and night shifts available daily for nursing interns during the training year? | 81 | 28.9 | 48 | 17.1 | 83 | 29.6 | 53 | 18.9 | 12 | 4.3 | 3 | 1.1 | **M** |
| 17 | How would you feel if there are one uniform and identification cards available and specified for nursing interns during the training year? | 44 | 15.7 | 75 | 26.8 | 92 | 32.9 | 46 | 16.4 | 11 | 3.9 | 12 | 4.3 | **Q** |
| 18 | How would you feel if the training system of nursing internship program starts with detailed orientation to clinical settings before each training area? | 30 | 10.7 | 65 | 23.2 | 132 | 47.1 | 35 | 12.5 | 15 | 5.4 | 3 | 1.1 | **M** |
| 19 | How would you feel if nursing internship program includes accessible accommodation or discount on the fees of accommodation for expatriates? | 114 | 40.7 | 41 | 14.6 | 39 | 13.9 | 68 | 24.3 | 12 | 4.3 | 6 | 2.1 | **A** |
|  | **Total training environment** | **33** | **11.8** | **32** | **11.4** | **89** | **31.8** | **111** | **39.6** | **15** | **5.4** | **0** | **0.0** | **O** |

**Note. O: One–dimensional, Q: Questionable, I: Indifferent, M: Must–be, A: Attractive, R: Reversal**

**Table (SUP 4): Distribution of the studied cases according to interns’ support Theme
(n = 280)**

| **Q** | **Interns support** | **A** | | **I** | | **M** | | **O** | | **Q** | | **R** | | **Category** |
| --- | --- | --- | --- | --- | --- | --- | --- | --- | --- | --- | --- | --- | --- | --- |
|  |  | **No.** | **%** | **No.** | **%** | **No.** | **%** | **No.** | **%** | **No.** | **%** | **No.** | **%** |  |
| 20 | How would you feel if the training system of nursing internship program includes available line of communication 24 hours with faculty members responsible for internship affairs? | 156 | 55.7 | 36 | 12.9 | 15 | 5.4 | 52 | 18.6 | 9 | 3.2 | 12 | 4.3 | **A** |
| 21 | How would you feel if the training system of nursing internship program includes available grievance procedure (nursing interns without fear of retribution? | 47 | 16.8 | 48 | 17.1 | 127 | 45.4 | 30 | 10.7 | 12 | 4.3 | 16 | 5.7 | **M** |
| 22 | How would you feel if there is a policy to prevent exploitation of nursing interns in the training settings? | 21 | 7.5 | 53 | 18.9 | 150 | 53.6 | 27 | 9.6 | 11 | 3.9 | 18 | 6.4 | **O** |
| 23 | How would you feel if complaints of nursing interns are discussed with transparency during the training year | 27 | 9.6 | 37 | 13.2 | 157 | 56.1 | 35 | 12.5 | 12 | 4.3 | 12 | 4.3 | **M** |
| 24 | How would you feel if the mentorship program is applied in the training system of nursing interns? | 131 | 46.8 | 72 | 25.7 | 12 | 4.3 | 45 | 16.1 | 15 | 5.4 | 5 | 1.8 | **A** |
|  | **Total interns support** | **59** | **21.1** | **34** | **12.1** | **22** | **7.9** | **142** | **50.7** | **14** | **5.0** | **9** | **3.2** | **O** |

**Note. O: One–dimensional, Q: Questionable, I: Indifferent, M: Must–be, A: Attractive, R: Reversal**

**Table (SUP 5): Distribution of the studied cases according to preceptorship Theme (n = 280)**

| **Q** | **Preceptorship** | **A** | | **I** | | **M** | | **O** | | **Q** | | **R** | | **Category** |
| --- | --- | --- | --- | --- | --- | --- | --- | --- | --- | --- | --- | --- | --- | --- |
|  |  | **No.** | **%** | **No.** | **%** | **No.** | **%** | **No.** | **%** | **No.** | **%** | **No.** | **%** |  |
| 25 | How would you feel if the training system of nursing internship program includes preceptors from nursing alumnae in each unit in clinical settings? | 120 | 42.9 | 55 | 19.6 | 21 | 7.5 | 59 | 21.1 | 17 | 6.1 | 8 | 2.9 | **A** |
| 26 | How would you feel if preceptors are available during the three shifts for education and mentoring? | 105 | 37.5 | 60 | 21.4 | 56 | 20.0 | 39 | 13.9 | 9 | 3.2 | 11 | 3.9 | **Q** |
| 27 | How would you feel if preceptors focus changed from just attendance monitoring to on-job training and clinical teaching? | 99 | 35.4 | 94 | 33.6 | 36 | 12.9 | 29 | 10.4 | 7 | 2.5 | 15 | 5.4 | **A** |
|  | **Total preceptorship** | **84** | **30.0** | **49** | **17.5** | **14** | **5.0** | **111** | **39.6** | **17** | **6.1** | **5** | **1.8** | **O** |

**Note. O: One–dimensional, Q: Questionable, I: Indifferent, M: Must–be, A: Attractive, R: Reversal**

**Table (SUP 6): Distribution of the studied cases according to incentives and motivation Theme (n = 280)**

| **Q** | **Incentives and motivation** | **A** | | **I** | | **M** | | **O** | | **Q** | | **R** | | **Category** |
| --- | --- | --- | --- | --- | --- | --- | --- | --- | --- | --- | --- | --- | --- | --- |
|  |  | **No.** | **%** | **No.** | **%** | **No.** | **%** | **No.** | **%** | **No.** | **%** | **No.** | **%** |  |
| 28 | How would you feel if the training system of nursing internship program includes achievement certificate to be awarded after each training area? | 134 | 47.9 | 38 | 13.6 | 14 | 5.0 | 62 | 22.1 | 15 | 5.4 | 17 | 6.1 | **A** |
| 29 | How would you feel if there is specified health insurance package available for nursing interns during the training year? | 46 | 16.4 | 79 | 28.2 | 99 | 35.4 | 29 | 10.4 | 9 | 3.2 | 18 | 6.4 | **R** |
| 30 | How would you feel if the training system of nursing internship program includes monthly monetary reward ((مكافاة مالية for the best performers in each area? | 113 | 40.4 | 56 | 20.0 | 25 | 8.9 | 66 | 23.6 | 17 | 6.1 | 3 | 1.1 | **A** |
| 31 | How would you feel if there is a valued salary with regular bonus increase for nursing interns during the training year? | 37 | 13.2 | 60 | 21.4 | 123 | 43.9 | 33 | 11.8 | 18 | 6.4 | 9 | 3.2 | **M** |
|  | **Total incentives and motivation** | **58** | **20.7** | **27** | **9.6** | **15** | **5.4** | **159** | **56.8** | **18** | **6.4** | **3** | **1.1** | **O** |

**Note. O: One–dimensional, Q: Questionable, I: Indifferent, M: Must–be, A: Attractive, R: Reversal**

**Table (SUP 7): Distribution of the studied cases according to autonomy Theme (n = 280)**

| **Q** | **Autonomy** | **A** | | **I** | | **M** | | **O** | | **Q** | | **R** | | **Category** |
| --- | --- | --- | --- | --- | --- | --- | --- | --- | --- | --- | --- | --- | --- | --- |
|  |  | **No.** | **%** | **No.** | **%** | **No.** | **%** | **No.** | **%** | **No.** | **%** | **No.** | **%** |  |
| 32 | How would you feel if the training system of nursing internship program offers opportunity for nursing interns to participate in the different committees of the training settings? | 134 | 47.9 | 77 | 27.5 | 9 | 3.2 | 40 | 14.3 | 9 | 3.2 | 11 | 3.9 | **A** |
| 33 | How would you feel if the training system of nursing internship program adopts a peer review assessment strategy during the training year? | 145 | 51.8 | 69 | 24.6 | 18 | 6.4 | 27 | 9.6 | 12 | 4.3 | 9 | 3.2 | **A** |
| 34 | How would you feel if there is a nursing internship advocacy committee that advocate for the rights of nursing interns during the training year? | 166 | 59.3 | 51 | 18.2 | 15 | 5.4 | 30 | 10.7 | 15 | 5.4 | 3 | 1.1 | **Q** |
| 35 | How would you feel if the training system of nursing internship program offers avenues for nursing interns to continue their postgraduate studies? | 150 | 53.6 | 61 | 21.8 | 17 | 6.1 | 35 | 12.5 | 14 | 5.0 | 3 | 1.1 | **A** |
|  | **Total autonomy** | **138** | **49.3** | **68** | **24.3** | **6** | **2.1** | **56** | **20.0** | **12** | **4.3** | **0** | **0.0** | **A** |

**Note. O: One–dimensional, Q: Questionable, I: Indifferent, M: Must–be, A: Attractive, R: Reversal**
